# Systematic analysis and mechanistic investigation of cardiac adverse events associated with antibody–drug conjugates using FAERS database

**DOI:** 10.1097/JS9.0000000000003314

**Published:** 2025-09-02

**Authors:** Xiaohan Zheng, Yongzhen Song, Yong Cao, Xuan Zhang, Kaiyuan Ge, Qi Zhang, Yaru Wang, Huimin Zhang, Shi Li, Lei Cao, Liuying Wang

**Affiliations:** a Department of Biostatistics, School of Public Health, Harbin Medical University, Harbin, China; b School of Health Management, Harbin Medical University, Harbin, China

**Keywords:** adverse drug events, antibody–drug conjugate, biological mechanisms, cardiac, FAERS

## Abstract

**Background:**

Antibody–drug conjugate (ADC), combining monoclonal antibodies with cytotoxic payloads through covalent linkers, is leading a new era of targeted cancer therapy. Despite its therapeutic success, cardiotoxicity has been a recognized concern in early approved ADCs such as trastuzumab, yet the cardiac safety profiles and mechanisms of recently approved ADCs remain poorly characterized. Assessing the nature of their cardiac events using the FDA Adverse Event Reporting System (FAERS) database is vital for risk assessment in surgical oncology. This study aimed to analyze cardiac adverse events (cAEs) related to ADCs in the FAERS database to detect cardiac risk signals, characterize clinical patterns, and investigate mechanistic pathways, ultimately informing risk mitigation strategies in clinical practice.

**Materials and methods:**

This systematic study analyzed FAERS data (Jan 2019–Sep 2023) for ADC-related adverse events (AEs). AEs were standardized using MedDRA terminology. Disproportionality analysis using the reporting odds ratio (ROR) method was performed to identify ADC-related cAEs. Potential risk factors were evaluated through logistic regression analyses. To understand the molecular basis, TCGA transcriptome data were analyzed to explore mechanisms of ADC-related cAEs.

**Results:**

cAEs comprised 11.77% (range: 8.63–23.50%) of all ADC-related reports from 2019 to 2023. A total of 49 cAE categories were identified. The mean age of reports with ADC-related cAEs was 60 (±13) years with 8.67% of reports having a fatal outcome. Breast cancer indications dominated. Combining ADCs with dexamethasone significantly reduced the risk of cardiac failure (ROR decreased from 3.18 to 0.85). ADC-related cAEs correlated with dysregulated HSP70 binding (*R* = 0.82, *P* = 4.66e-4) and heat shock protein pathways.

**Conclusions:**

This study underscores the importance of recognizing and managing the cAEs associated with ADC therapy. Our findings provide a nuanced understanding of the burden, risk factors, and potential biological mechanisms of ADC-related cAEs, which can inform clinical decision-making and guide the development of safer and more effective ADC therapies.

**HIGHLIGHTS**

- Systematic analysis of antibody–drug conjugate (ADC)-related cardiac adverse events (cAEs) using FAERS database.
- Female and older patients have higher cAE risk, notably in breast cancer.
- Dexamethasone with ADCs lowers cardiac failure risk.
- Over 50% of cAEs occur within 3 months, varying by gender and age.
- HSP70 binding pathways linked to cAEs, suggesting therapeutic targets.

## Introduction

Antibody–drug conjugate (ADC) is typically a complex formed by covalently coupling a small molecule drug (payload) with a monoclonal antibody through a linker[1]. ADCs combine monoclonal antibodies’ specificity with cytotoxic payloads, enabling targeted cancer cell elimination[2]. In 2000, the U.S. Food and Drug Administration (FDA) ushered in the era of targeted cancer therapy with the approval of the first ADC drug, Mylotarg, specifically designed for adults with relapsed cluster of differentiation CD 33-positive acute myeloid leukemia[3]. Since 2019, the development of ADCs has displayed a definitive upward trajectory. Polivy, Padcev, and Enhertu were approved by the FDA for treatment of B-cell lymphoma, urothelial tumor, and HER2-positive breast cancer within the year[4]. In the next 3 years, Trodelvy, Akalux, Zynlonta, RC48, Tivdak, and Elahere are expected to be launched successively, targeting triple-negative breast cancer, head and neck cancer, large B-cell lymphoma, gastric cancer, urothelial carcinoma, cervical cancer, and ovarian cancer. The expanding targets and indications of ADCs suggest their potential to complement or replace conventional chemotherapy in specific cancers[5].

While ADC drugs are gaining increasing attention in cancer therapy, their potential to induce a diverse range of adverse reactions merits significant consideration. The most frequently encountered severe side effect (of grade 3 or above), hematotoxicity, which manifests as neutropenia, thrombocytopenia, leukopenia, and anemia, is likely attributed to the premature release of cytotoxic agents into the bloodstream, coupled with hepatotoxicity and gastrointestinal reactions[6]. Furthermore, the immune response elicited by the antibody component of ADC may contribute to secondary injuries, thereby leading to nephrotoxicity[7]. The cardiac adverse reactions, encompassing cardiotoxicity, cardiac dysfunction, and reduced ventricular compliance, associated with cancer therapies, can significantly hamper the continuation of optimal treatment, posing life-threatening risks or prolonged morbidity for patients[8]. Cardiac adverse reactions are a category of ADC-related side effects that occur infrequently but can vary widely in severity” to improve clarity and conciseness. For example, a study by Xu *et al* found that the arrhythmia of ADCs is rare but hold the potential for life-threatening consequences[9]. In AML-19, the safety and efficacy evaluation of gemtuzumab ozogamicin showed that among the Grade ≥ 3 adverse events (AEs) observed in over 5% of patients, cardiac reactions stood out at 6%[10]. Given the growing interest in ADCs for cancer therapy and the potentially severe consequences of cardiac adverse reactions, despite their low incidence, further research into the occurrence, mechanisms and management strategies of cardiac system adverse reactions of ADCs is crucial. This will enhance the safety profile of ADC treatments and enable patients to receive more durable and effective therapeutic options^[9,10]^. However, there is still a notable absence of a comprehensive study and consolidated summary of cardiac adverse events (cAEs) associated with ADCs. The actual clinical and epidemiological significance of these uncommon AEs is often more accurately gauged in real-world data sources, such as the FAERS database, rather than solely relying on registration trials[11].

In summary, given the potential clinical significance of cardiac adverse reactions, a thorough analysis is imperative to delve into the association between ADCs and cAEs, as well as the factors that influence them. This study leveraging the FAERS database reports from 2019 to 2023, cAEs were tallied, and a disproportionality analysis was undertaken to pinpoint cardiac adverse reactions and uncover potential influencing factors and biological mechanisms closely linked to ADCs. This study aims to furnish a profound and comprehensive understanding of cAEs associated with ADCs, serving as a valuable reference for clinical practice. Although artificial intelligence (AI) was not used in this study, we have ensured compliance with the TITAN 2025 guidelines on transparency and reporting of AI-related research[12].

## Methods

### Dataset acquisition and processing

The FDA Adverse Event Reporting System (FAERS) is a publicly accessible database comprising AE reports submitted by healthcare professionals, manufacturers, and consumers[13]. It includes seven datasets: demographic information (DEMO), drug details (DRUG), adverse reaction specifics (REAC), patient outcomes (OUTC), report sources (RPSR), medication timelines (THER), and drug indications (INDI)^[14,15]^. Outcomes recorded in the FAERS database were coded using standard FDA outcome abbreviations, including death (DE), life-threatening (LT), hospitalization (HO), disability (DS), congenital anomaly (CA), required intervention to prevent permanent impairment (RI), and other medically important events (OT).We retrieved ADC-related reports from the FAERS Public Dashboard (2019Q1–2023Q3) using the following keywords: enhertu, polivy, padcev, trodelvy, tivdak, akalux, zynlonta, RC48, and elahere. Only cases with ADCs listed as “primary suspect” drugs were included.

**Figure 1.**
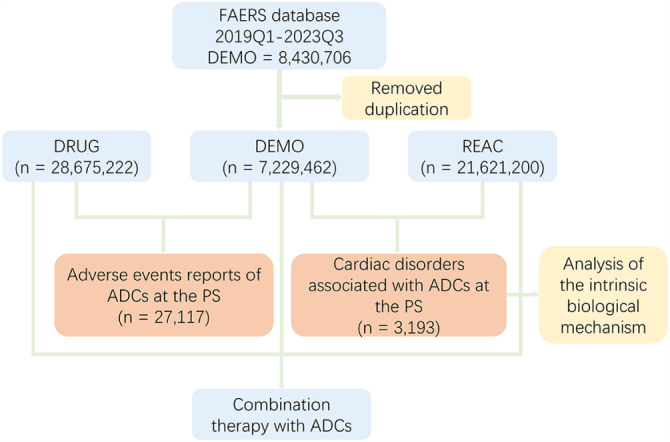
The FAERS database’s pipeline flowchart for screening cardiac disorders associated with ADCs.

To address duplicate entries, we followed FDA deduplication guidelines[16]. For identical CASEIDs, the report with the latest FDA_DT (submission date) was retained. If CASEID and FDA_DT matched, the entry with the higher PRIMARYID was selected. Subsequently, we screened reports by comparing sex, age, country, event date, adverse reactions, drugs, and indications to ensure uniqueness (Fig. 1). This process yielded 27 117 unique ADC-related reports.

cAEs were identified using the Medical Dictionary for Regulatory Activities (MedDRA v25.0). We focused on Preferred Terms (PTs) classified under the System Organ Class (SOC) “cardiac disorders,” ensuring clinically significant events were analyzed.

### Signal mining

In the field of pharmacovigilance, disproportionality analysis plays a fundamental role as a screening tool to identify potential relationships between a specific AE and a drug. Once identified, these potential associations can be further explored through clinical evaluations of individual case reports[17]. The reporting odds ratio (ROR) serves as a metric to compare the likelihood of reporting a particular AE for a drug, relative to all other events, with the likelihood of reporting any AE for other drugs in the FAERS database[18]. In our study, we utilized the ROR to detect signals of cAEs in ADC reports. By leveraging the comprehensive adverse reaction reports from the FAERS database as a reference, we conducted a disproportionality analysis to assess the potential association between cardiac adverse reactions and ADCs by calculating the ROR[18]. This analysis enables us to evaluate potential associations between specific AEs and drugs, based on a comparison of the observed versus expected number of AEs for each drug–event pair[19].

The following formula was used to calculate the ROR and 95% confidence interval (CI):

$$ROR=\frac{a/c}{b/d}$$

$$95\%CI=e^{ln(ROR)\pm 1.96\sqrt{\frac{1}{a}+\frac{1}{b}+\frac{1}{c}+\frac{1}{a}}}$$

The cardiac adverse reaction signal was deemed valid and closely linked to ADC therapy when there were no fewer than three reports of cAEs, and the lower boundary of the 95% CI for the ROR surpassed one[20]. Consequently, PTs of cardiac disorders satisfying these criteria were classified as ADC-related cAEs. ADC reports that involved the occurrence of ADC-related cAEs were selected for further evaluation (*N* = 3193).

### Descriptive analysis

We performed a descriptive analysis of the clinical characteristics of reports with ADC-related cAEs after screening, including sex, age, age-group, country, outcome, latest year of FDA acceptance, ADC treatment strategy, treatment indication, case priority, report type, and other clinical characteristics. The onset time of ADC-related cAEs was defined as the interval between EVENT_DT (date of AE occurrence) and START_DT (date of ADCs initiation). In addition, reports with data input errors (EVENT_DT earlier than START_DT) or inaccurate dates were excluded[15]. Cumulative distribution curves were used to present event-to-onset information for ADC-related cAEs in different groups.

### Calculating the enrichment scores of biological pathways in TCGA pan-cancer

We obtained transcriptome data in count format from the University of California, Santa Cruz (UCSC) Xena database[21] for 26 cancer species within the TCGA project. Subsequently, we converted the count expression matrix into a transcripts per million matrix[22]. Single-sample gene set enrichment analysis (ssGSEA) was then performed at the pan-cancer level using the GSVA package[21]. This involved calculating the enrichment scores of Gene Ontology biological pathways[23] within the samples of each cancer type. The enrichment score of a specific gene set reflects the activity level of the corresponding biological process, indicating coherent up- or downregulation of the gene set members[24]. The average enrichment score within a given pathway represents the activation level of that pathway in the respective type of cancer[25]. Our objective is to examine the correlation between ADC-related cardiovascular AEs ROR and the activation level of biological pathways at the pan-cancer level, with the aim of identifying potential biological mechanisms associated with ADC-related cardiovascular AEs.

### Statistical analysis

In this study, event-free probabilities for the time to onset of ADC-related cAEs were estimated via the Kaplan–Meier method. The log-rank test was applied to compare the median time to onset among distinct groups experiencing ADC-related cAEs. Since all cases had experienced the event of interest with fatal outcomes occurring following ADC-related cAEs, censoring or competing risk factors were not considered in the analysis. Categorical variable differences between groups and statistical significance in disproportional analyses were assessed using the Chi-square test or Fisher’s exact test, depending on the data characteristics. Exposure factors for ADC-related cAEs were identified as ADC treatment strategy, age, sex and outcome. To quantify the crude effects of these exposures on the occurrence of ADC-related cAEs, a univariate logistic regression analysis was performed, resulting in crude odds ratios (ORs). However, to account for the influence of multiple exposures and potential confounders, a multivariate logistic regression analysis was further conducted to estimate adjusted ORs. Additionally, a correlation analysis was performed between pan-cancer level ADC-related cAEs RORs and enrichment scores of biological pathways, employing Spearman’s rank correlation coefficient. Samples with missing values for each clinical characteristic were excluded from the statistical analysis. For all statistical tests, a two-sided *P*-value of less than 0.05 was considered statistically significant, indicating strong evidence against the null hypothesis. Additionally, CIs for ORs that did not include 1 were interpreted as statistically significant associations. All statistical analyses and visualization were conducted using R software (version 4.3.2, https://www.r-project.org/). The work has been reported in line with the REMARK criteria[26].

## Results

***Scanning for ADC-related cAEs among ADC users in the FDA AEs reporting system, 2019–2023***

**Figure 2.**
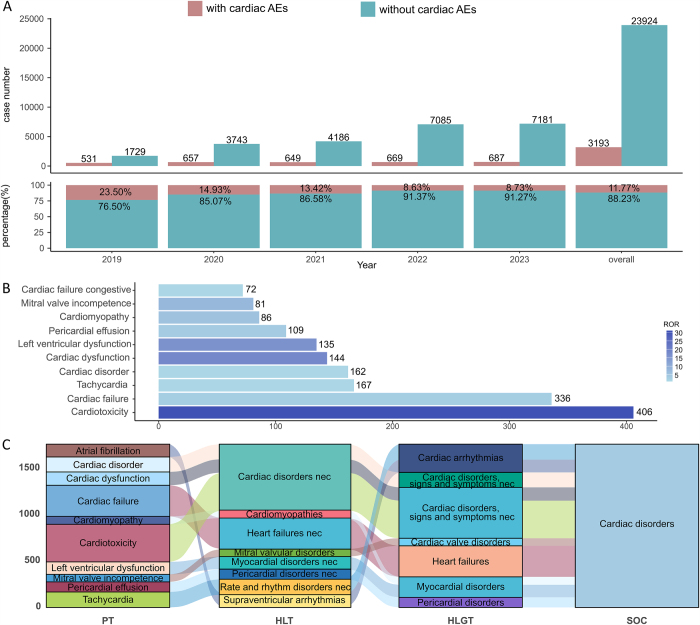
Overview of ADC-associated cardiac adverse events in FAERS (2019Q1–2023Q3). (A) Bar plots showing the number and proportion of ADCs reports with vs without cardiac adverse events. (B) Top 10 most frequently reported cardiac adverse events across different ADC treatment strategies. (C) UpSet plot of ADC-related cAEs across different cancers.

We first investigated the occurrence of cAEs among patients treated with ADCs in the FAERS database from 2019Q1 to 2023Q3. The detailed data processing procedure is shown in Figure 1. The proportional bar plot below shows the amount of ADC reports with cAEs versus ADC reports without cAEs in the FAERS database during 2013 and 2023, as well as the overall situation, in 2019, cAEs accounted for a relatively high 23.50% of total cases. However, since then, the percentage has gradually declined, with 2020 seeing a drop to 14.93%, and further reductions in 2021 to 13.42%, 2022 to 8.63%, and 2023 to 8.73%. Despite the overall downward trend, cAEs continue to represent a significant share of all adverse reactions reported, averaging out to 11.77% across all years considered (Fig. 2A). Overall, the proportion of cAEs under ADCs suggests that it is a nonnegligible part of the AEs associated with ADCs. After compiling the categories of cAEs and their respective case counts in ADC reports, we conducted a disproportionality analysis. This involved calculating the ROR for PTs with at least three cases related to the aforementioned cAEs, using the comprehensive FAERS database as a benchmark. Ultimately, 48 PTs for cardiac disorders that exhibited a strong association with ADC treatment in the overall context were categorized as ADC-related cAEs [Supplemental Digital Content Table 1, Available at, http://links.lww.com/JS9/E977]. Among these, cardiotoxicity, cardiac failure, tachycardia, cardiac disorder, cardiac dysfunction, atrial fibrillation, left ventricular dysfunction, pericardial effusion, cardiomyopathy, mitral valve incompetence and cardiac failure congestive comprised the top ten categories of ADC-related cAEs with the highest case counts (Fig. 2B). Specifically, cardiotoxicity stood out as the ADC-related cAE with the highest number of cases and exhibited significant ROR signals. The top five most frequently reported cases of ADC-related cAEs were present across all cancers. We extended the PT-level analysis across key subgroups, including sex, age group, ADC drug type, and cancer type. For each subgroup, we calculated the ROR values of cardiac-related PTs consistent with the criteria applied in the overall analysis. The resulting subgroup-specific profiles of ADC-related cAEs are summarized in Supplemental Digital Content Table 3, Available at, http://links.lww.com/JS9/E979. Notably, cardiotoxicity, cardiac dysfunction, and left ventricular dysfunction consistently ranked among the top PTs across most subgroups. These subgroup analyses not only support the robustness of our primary findings but also help identify patterns that may be specific to certain populations or treatment contexts. We also found that breast cancer and gastric cancer had unique cAEs. Figure 2C depicts the association of the PTs related to ADC-related cAEs with other hierarchical levels within the MedDRA, where the primary SOC is cardiac disorder.

### Descriptive analysis of cases with ADC-related cAEs

After screening the reports of ADCs in the FAERS database, we identified 27 117 cases pertaining to ADCs. Among these, 3193 reports were distinctly linked to cAES. We then statistically described the clinical characteristics of these cases in Table 1. Notably, among the available data for cardiac-related AE reports as well as other AE reports, a significantly higher percentage of females was documented. Specifically, females accounted for 90.63% of cAE reports and 80.74% of other AE reports, whereas males comprised only 9.37% and 19.22%, respectively. Patients with cAEs were older than those with other AEs (60.12 ± 13.18 vs. 58.92 ± 13.86 years, *P* = 0.0022). With regard to weight, the majority of patients with cardiac AEs (78.84%) and other AEs (82.26%) fell into the weight range of less than 80 kg. However, a higher percentage of patients with cardiac AEs (16.20%) belonged to the 80–100 kg weight range compared to those with other AEs (12.13%). The outcomes of the AE reports varied significantly between the cardiac and other AE groups, the majority of cases in both groups were nonfatal. However, a higher proportion of fatal cases was observed in the other AE group (11.20%) compared to the cardiac AE group (7.84%).

**Table 1:** Clinical characteristics of reports with ADCs from the FAERS database

Characteristics	ADC-related cardiac AE reports (*n* = 2361)	ADC-related other AE reports (*n* = 24 756)	*P* value
Gender, *n* (%)			<0.0001
Female	1596 (90.63)	15 646 (80.74)	
Male	165 (9.37)	3724 (19.22)	
UNK	0 (0.00)	8(0.04)	
Age (years)			0.0022
*n* (Missing)	1272 (1089)	12 527 (12 229)	
Mean ± SD	60.12 ± 13.18	58.92 ± 13.86	
Weight (kg), *n* (%)			0.0140
<80	477 (78.84)	5201 (82.26)	
80≤and≤100	98 (16.20)	767 (12.13)	
>100	30 (4.96)	355 (5.61)	
Outcomes, *n* (%)			<0.0001
CA	0 (0.00)	12 (0.07)	
DE	185 (8.67)	2773 (15.47)	
DS	38 (1.78)	195 (1.09)	
HO	437 (20.48)	4650 (25.95)	
LT	88 (4.12)	381 (2.13)	
OT	1384 (64.85)	9884 (55.16)	
RI	2 (0.09)	25 (0.14)	
Fatality, *n* (%)			<0.0001
Nonfatal	2176 (92.16)	21 983 (88.80)	
Fatal	185 (7.84)	2773 (11.20)	

**Figure 3.**
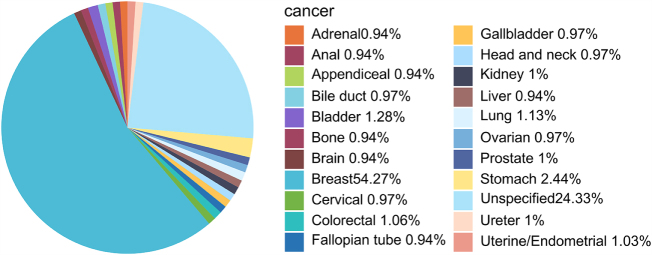
The pie chart of the proportional composition of the patient’s cancer original sites.

Among the cases involving cAEs related to ADC, the majority were those with indications for breast cancer, accounting for 54.27% of the total (Fig. 3). Considering the potential impact of this large proportion on the overall results, we further conducted subgroup analyses by separately examining cases with and without breast cancer. These additional analyses, presented in the Supplemental Digital Content Material 1, Available at, http://links.lww.com/JS9/E981 and Supplemental Digital Content Material 2, Available at, http://links.lww.com/JS9/E982, were performed to assess the robustness of the findings and ensure that key observations were not disproportionately influenced by the predominance of breast cancer cases. A comprehensive list of common cAEs across all cancer types is presented in Table 2, while Table 3 highlights the specific AEs associated with breast cancer. Table 4 provides detailed information on patients who experienced cardiac aneurysm, a specific cAE associated with trastuzumab warranting further investigation.

**Table 2:** Common ADC-related cAEs across all cancer types

PT	Events	ROR (95% CI)
Cardiotoxicity	406	30.99 (27.94, 34.36)
Cardiac dysfunction	144	18.00 (15.19,21.31)
Ventricular hypokinesia	45	11.55 (8.56,15.57)
Mitral valve incompetence	81	7.82 (6.27,9.75)
Cardiac failure	336	3.18 (2.85,3.54)
Angina pectoris	54	1.77 (1.35,2.31)
Tachycardia	167	1.47 (1.26,1.71)
Cardiac disorder	162	1.39 (1.19,1.62)

**Table 3:** Breast cancer-specific ADC-related cAEs

PT	Events	ROR (95% CI)
Right ventricular dysfunction	13	12.77(5.29, 30.82)
Cardiac aneurysm	5	39.30 (4.59, 336.38)
Ventricular remodeling	5	39.30 (4.59, 336.38)
Myocardial injury	11	8.65 (3.67, 20.36)
Systolic dysfunction	12	5.24 (2.52,10.88)
Pericarditis	19	3.93 (2.27, 6.82)
Cardiac failure acute	14	4.08 (2.14, 7.77)
Cardiac hypertrophy	5	6.55 (2.00, 21.46)
Toxic cardiomyopathy	5	4.37 (1.46, 13.03)
Tricuspid valve disease	2	15.72 (1.43, 173.35)
Cardiac ventricular disorder	2	15.72 (1.43, 173.35)
Cardiogenic shock	13	2.43 (1.31, 4.53)
Hypertensive heart disease	4	3.93 (1.18, 13.05)
Ischemic cardiomyopathy	2	7.86 (1.11, 55.80)
Myopericarditis	2	7.86 (1.11, 55.80)
Cardiac ventricular thrombosis	4	3.49 (1.08, 11.34)

**Table 4:** Detailed information of patients with cardiac aneurysm as a breast cancer-specific ADC-related cAE

Case id	Age	Gender	Weight (kg)	Drug name	Outcome	GetDataYear
18702190	88	F	NA	Trastuzumab	HO	21Q1
18784043	88	F	NA	Trastuzumab	HO	21Q1
19263219	NA	NA	NA	Trastuzumab	HO	21Q2
19429932	88	F	NA	Trastuzumab	HO	21Q2
21766930	74	F	58.2	Trastuzumab	HO	22Q4

### Time to onset analysis

**Figure 4.**
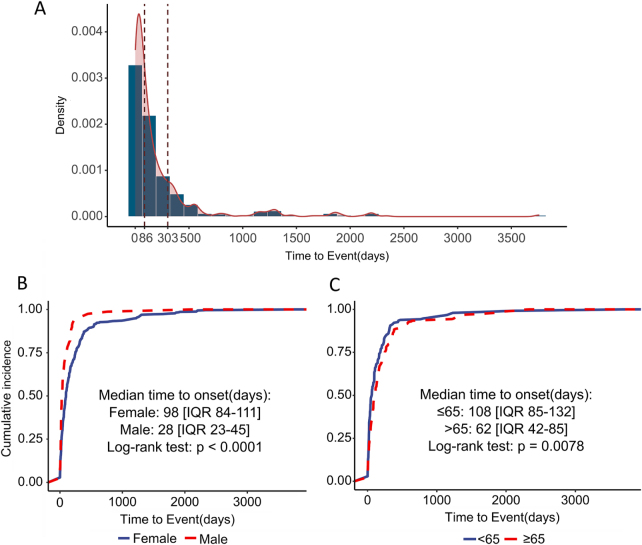
The statistical chart of ADC-related cAEs reaction onset time. (A) Cumulative distribution curve of overall onset time following ADC treatments. (B, C) Cumulative distribution curves of onset time stratified by gender (B) and by year (C).

Over half of the cases involving ADC-related cAEs emerged in the initial 3 months following the commencement of ADC treatments, while over 80% occurred within the ten months of initiating such treatments (Fig. 4A). The median onset time was significantly shorter in males compared to females (Days: 28 vs. 98, *P* < 0.05) (Fig. 4B). Additionally, patients above 65 years old had a median onset time of 20 days longer than those under 65 years old (Days: 118 vs. 98, *P* < 0.05) (Fig. 4C).

### Influencing factors for ADC-related cAEs

**Figure 5.**
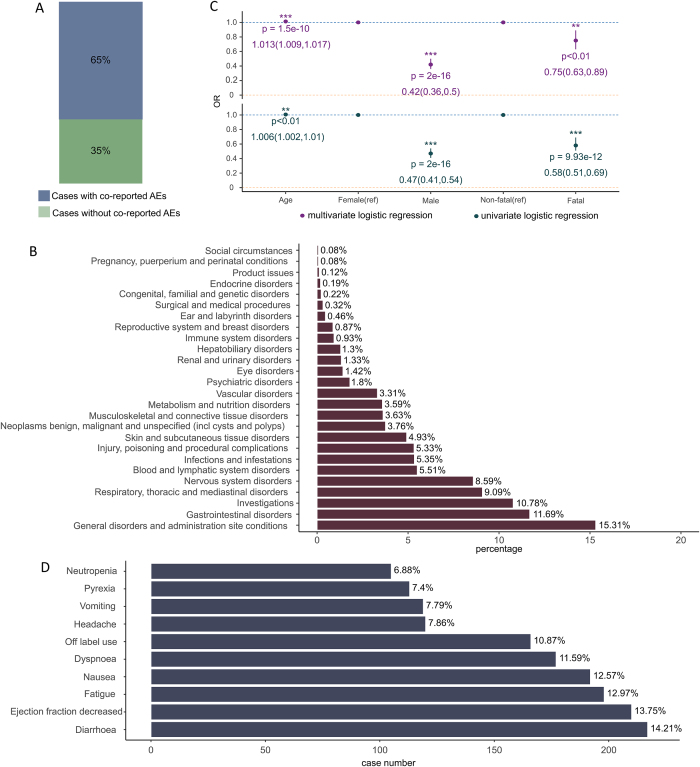
Factors influencing ICI-related cardiac adverse events. (A) Proportion of cases with vs without co-reported adverse events among ADC-related cAE cases. (B) Distribution of co-reported adverse events by SOC. (C) Forest plot of univariate (green) and multivariate (purple) logistic regression results for factors associated with ADC-related cAEs. OR indicates odds ratio. *p < 0.05; **p < 0.01; ***p < 0.001; (D) Top 10 PTs of co-reported adverse events.

We have additionally examined co-reported AEs that may act as influencing factors for ADC-related cAEs. Among the cases with ADC-related cAEs that we screened, 65% of cases were accompanied by the occurrence of other AEs, while 35% of cases had only ADC-related cAEs (Fig. 5A). Among cases with other concomitant AEs, general disorders and administration site conditions were the most frequently reported, occurring in approximately 15.31% of the cases. Gastrointestinal disorders and investigations followed closely, accounting for 11.69% and 15.31% of the total AE cases, respectively (Fig. 5B). Additionally, diarrhea, ejection fraction decreased, and fatigue are the three most common concomitant AEs at the PT level (Fig. 5D).

We further explored factors that might influence the occurrence of ADC-related cAEs by univariate logistic regression analysis and multivariate logistic regression analysis based on the total ADC reports (Fig. 5C). First, through univariate logistic regression analysis, we found that age was a significant factor, with an OR value of 1.006 and a 95% CI of (1.002, 1.01), indicating a slight increase in the risk of ADC-related cAEs with advancing age. Additionally, gender was also an important factor, as males had a lower risk of ADC-related cAEs compared to females, with an OR value of 0.47 and a 95% CI of (0.41, 0.54), suggesting that the risk for male patients is approximately half that of female patients. Compared to nonfatal events, fatal events had a lower risk of ADC-related cAEs, with an OR value of 0.58 and a 95% CI of (0.51, 0.69). In the multivariate logistic regression analysis, we discovered that age, gender, and fatality remained significant factors. The OR value for age increased to 1.013, with a 95% CI of (1.009, 1.017), further confirming the positive correlation between age and the risk of ADC-related cAEs. The OR value for gender also slightly decreased to 0.42, with a 95% CI of (0.36, 0.5), while the OR value for fatality changed to 0.75, with a 95% CI of (0.63, 0.89), indicating that in the multivariate model, fatal events still had a lower risk of ADC-related cAEs compared to nonfatal events.

### Analysis of related cAEs in combination therapy with ADCs

**Figure 6.**
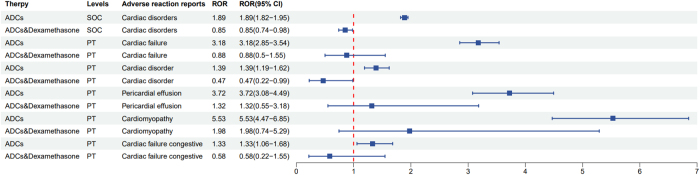
The signal distribution of ADCs alone and the combination of ADCs and dexamethasone.

We retrieved the drug combinations associated with ADCs from the FAERS database and performed a disproportionate analysis of adverse reactions for those drug combinations with a higher frequency of use. We found that the combination of ADCs and dexamethasone can reduce the risk of heart disease (Fig. 6), the ROR for cardiac failure is 3.18 for ADCs alone, whereas it drops to 0.85 when combined with Dexamethasone. Similarly, the ROR for pericardial effusion decreases from 1.39 to 0.47, and for cardiomyopathy, it decreases from 3.72 to 1.32 when ADCs are administered concomitantly with Dexamethasone. Baseline characteristics of patients (cases) with cardiac AEs reported for ADCs alone, and combination dexamethasone therapy are presented and compared in Supplemental Digital Content Table 2, Available at, http://links.lww.com/JS9/E978. The gender and age distributions were not statistically significant between those receiving ADCs alone and those receiving a combination of ADCs and dexamethasone but there was a statistically significant difference in weight distribution, with patients receiving the combination therapy tending to be heavier.

### Analysis of the intrinsic biological mechanism associated with ADC-related cAEs

**Figure 7.**
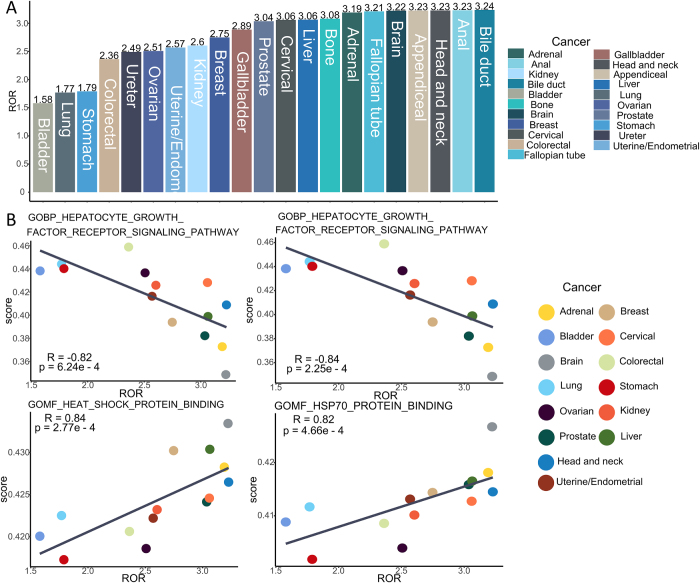
Assessment of the association between ADC-related cardiac adverse events and biological pathways. (A) Bar plot of RORs for ADC-related cAEs across 21 cancer types. (B) Correlation between RORs of ADC-related cAEs and ssGSEA pathway enrichment scores, analyzed using Spearman’s rank correlation.

We delved into the potential biological mechanisms behind the emergence of ADC-related cAEs (Fig. 7). At the pan-cancer level, the ROR of ADC-related cAEs varied depending on the cancer type. Specifically, bile duct cancer exhibited the highest ROR for ADC-related cAEs, standing at 3.24 (95% CI: 2.42–4.33), whereas bladder cancer showed the lowest ROR, measuring 1.58 (95% CI: 1.20–2.07). Upon integrating transcriptome data analysis across TCGA pan-cancers, we discerned a significant positive correlation between the pan-cancer level ADC-related cAE ROR and the binding affinity of heat shock protein (HSP) (*R* = 0.84, *P* = 2.77e-4) as well as HSP70 protein (*R* = 0.82, *P* = 4.66e-4). Conversely, a significant negative association was observed between the pan-cancer level ADC-related cAE ROR and the hepatocyte growth factor receptor signaling pathway (*R* = −0.82, *P* = 6.24e-4), as well as the calcium-dependent cysteine type endopeptidase activity (*R* = −0.84, *P* = 2.25e-4).

## Discussion

Despite the growing body of research on ADCs, their potential to induce cAEs remains insufficiently understood. Therefore, we conducted a comprehensive pharmacovigilance analysis using the FAERS database from 2019 to 2023, identifying associations between ADCs and cAEs, profiling clinical characteristics, and exploring potential biological mechanisms through TCGA transcriptome integration. Our findings provide a multidimensional framework for identifying vulnerable individuals, enabling early detection, and informing risk-adapted treatment strategies.

Our disproportionality analysis revealed 49 cAEs significantly associated with ADC exposure, representing a broad and concerning spectrum of cardiovascular toxicity. Notably, cardiotoxicity, cardiac failure, and cardiac dysfunction were the most frequently reported and strongly associated PTs, with cardiotoxicity exhibiting a markedly elevated ROR of 30.99. These signal strengths far exceed commonly used thresholds for safety concern and underscore the potential seriousness of ADC-induced cardiac events in post-marketing surveillance. Given the diversity and consistency of these associations, further exploration of at-risk populations and contributing factors is urgently needed.

Given the diverse indications of ADCs, we conducted subgroup analyses to evaluate whether certain populations exhibited disproportionately higher cardiac risk. Older patients showed a significantly higher risk, consistent with their elevated baseline cardiovascular burden and comorbidities[27]. Age-stratified disproportionality analyses (cutoff: >65 years) demonstrated consistent ROR elevations for key cardiac events across both elderly and younger subgroups, indicating that age amplifies but does not solely account for ADC-related cardiac risk. Male sex was consistently associated with a lower incidence of cAEs in multivariate analyses. This protective effect may relate to estrogen’s cardioprotective role in premenopausal women[28]. However, the average age of female patients experiencing ADC-related cAEs was 60 years, suggesting most were postmenopausal[29]. Notably, female patients also exhibited a longer latency period before cAE onset. These findings suggest a complex interplay of hormonal and molecular factors, including age and sex related hormonal differences and disruption of the neuregulin-1/HER2 signaling pathway, which plays a critical role in cardiomyocyte survival and myofibrillar maintenance^[30,31]^. Together, these observations underscore the need for intensified cardiac surveillance in older patients and prolonged monitoring in female patients, even after ADC therapy concludes.

Beyond demographic risk stratification, we further examined co-occurring symptoms and modifiable treatment factors that may offer opportunities for early detection and proactive clinical intervention. Diarrhea was frequently co-reported with cardiac events, present in 65% of cAE cases with additional adverse effects. This association is supported by both biological plausibility and clinical relevance: severe diarrhea can lead to electrolyte imbalances and dehydration – known contributors to cardiac dysfunction and failure^[32,33]^. Consequently, diarrhea during ADC treatment should prompt immediate assessment of electrolyte status and cardiac function. In real-world oncology practice, ADCs are frequently administered as part of combination regimens[34]. Our exploration of concomitant medications revealed that coadministration of dexamethasone significantly attenuated the risk of multiple cardiac events, including cardiac failure (ROR reduced from 3.18 to 0.85) and pericardial effusion (ROR reduced from 1.39 to 0.47). As a glucocorticoid, dexamethasone possesses anti-inflammatory and cardioprotective properties that may counteract ADC-induced myocardial stress and inflammation[35]. These findings suggest that dexamethasone co-treatment could represent a viable strategy for cardioprotection, particularly among high-risk individuals undergoing ADC therapy, and warrants further clinical validation.

While these clinical associations provide valuable insight into risk profiles and potential interventions, we further sought to elucidate the underlying molecular mechanisms driving ADC-related cardiac injury. This analysis revealed a strong positive correlation between cAE risk and the activation of HSP-related pathways, particularly HSP70 binding^[36,37]^. HSPs regulate proteostasis and cellular stress. Impaired HSP function can lead to misfolded protein accumulation and has been implicated in various forms of cardiac injury, especially in terminally differentiated cardiomyocytes with limited regenerative capacity[38]. Conversely, significant negative correlations were identified with protective pathways such as hepatocyte growth factor receptor (HGFR) signaling and calcium-dependent cysteine endopeptidase activity^[39,40]^, suggesting their disruption may also contribute to cardiac vulnerability. Additionally, emerging studies in cancer bioelectronics propose that electrophoretic and dielectrophoretic properties of tumor cells may influence systemic organ responses, including cardiac tissue[41]. Patients exhibiting dysregulated stress or impaired protective signaling may be particularly vulnerable to ADC-induced cardiac toxicity. Molecular profiling of such pathways may enable early identification of at-risk individuals and guide personalized preventive strategies. Furthermore, the identified pathways may inform future biomarker development and guide risk-adapted monitoring or cardioprotective interventions. This approach could ultimately support more personalized, mechanism-informed cardiac care in ADC-treated populations.

Given the predominance of breast cancer cases in our cohort, we conducted comprehensive subgroup analyses to ensure our findings represent genuine ADC class effects rather than breast cancer–specific phenomena. Importantly, when breast cancer cases were excluded, the core cardiac safety signals remained robust and statistically significant.

In the non-breast cancer subgroup, key AEs including cardiotoxicity, left ventricular dysfunction, and cardiac failure maintained statistical significance with effect sizes comparable to the overall population. Notably, this analysis revealed additional signals such as angina pectoris that were previously masked, suggesting that cancer type diversity may obscure certain safety concerns. Detailed information on patients with angina pectoris in the non-breast cancer cohort is presented in Supplemental Digital Content Table 4, Available at, http://links.lww.com/JS9/E980. Demographic risk patterns, including lower risk in males and delayed onset in younger and female patients, remained consistent and support the robustness of our findings across cancer types.

In comparison, patients with breast cancer showed a higher baseline risk of cardiac events and a longer time to onset. The median time to event in this group was 115.5 days, which was longer than in other cancer types. This delayed onset may reflect disease-related factors or differences in ADC regimens. Despite the higher rate, the types of cardiac events and their relative risks were similar to those in the full cohort. Age was the main risk factor in this group. The effect of sex was not significant, likely due to the small number of male patients with breast cancer. Notably, dexamethasone showed a consistent protective effect against cardiac events in both breast cancer and non-breast cancer patients. This suggests that it may help reduce heart-related risk regardless of cancer type.

To better understand these differences, we compared the rates of cardiac events across various cancer types. Several cancers, such as bile duct, anal, brain, prostate, and adrenal cancers, had cardiac event rates as high as or higher than breast cancer. In contrast, stomach and bladder cancers had lower rates. We also observed that patients with stomach cancer developed cardiac events much earlier than those with breast cancer. The median time to onset in stomach cancer was only 28 days. These differences highlight the importance of adjusting cardiac monitoring based on the specific cancer type and treatment setting.

Beyond clinical observations, we also examined possible biological explanations. In non-breast cancer patients, the key molecular pathways we identified earlier remained significantly correlated with cardiac risk. These included stress-related and survival pathways such as HSP70 and HGFR signaling. In addition, two new pathways were identified. One involved reduced cellular movement across blood vessels. The other was related to cell death triggered by oxidative stress. These findings suggest that damage to the heart may be caused by both protein stress and changes in the immune environment. This helps expand our understanding of the possible mechanisms behind ADC-related cardiac toxicity.

In summary, our comprehensive analysis delineates a multilayered risk profile for ADC-associated cardiotoxicity, integrating demographic susceptibility, clinical warning signs, treatment modifiers, and underlying molecular mechanisms. Subgroup analyses confirmed that these risks are not confined to breast cancer, with consistent cardiac safety signals observed across multiple tumor types, though with cancer-specific variations in onset timing and event spectrum. These findings underscore the importance of tailored cardiac monitoring strategies and proactive risk mitigation measures based on patient characteristics and cancer types. Incorporating cardiac surveillance, early symptom recognition, and protective intervention into routine clinical practice may improve the safety profile of ADC therapy and support more individualized, mechanism-informed patient care.

This study faces several limitations. First, the FAERS database, being a global spontaneous reporting system, inherently introduces biases due to factors like the ethnic and geographical distribution of reported cases, the timing of drug approvals and market access, varying levels of public awareness toward specific adverse reactions, and the incomplete capture of all serious AEs. Therefore, we could not obtain a causal relationship between ADCs and ADC-related cAEs, nor could we calculate the incidence of cAEs or ADC-related cAEs. Due to the high proportion of breast cancer cases among patients with ADC-related cAEs, we performed stratified analyses. The consistent safety patterns support the robustness of our findings. However, this imbalance may still lead to overrepresentation of breast cancer-specific signals, reflecting both clinical ADC usage and real-world reporting trends. Caution is warranted when generalizing these results to other tumor types. Second, our focus on the top three drug combinations by database record count for concurrent medication analysis might not fully mirror real-world drug combinations. A deeper dive into the coadministered drugs and their AE profiles is imperative for a comprehensive understanding of ADC-related cAEs in combination therapy. Lastly, the cardiac adverse reactions linked to ADCs in our study are yet to undergo clinical validation, highlighting a research gap in this domain. As a comprehensive case study with an exploratory nature, our observations regarding ADC-related cAEs necessitate confirmation through large-scale, prospective research endeavors.

## Conclusion

This study identified 49 cAEs significantly associated with ADCs through FAERS-based disproportionality analysis. Subgroup analyses further revealed that these cardiac risks extend beyond breast cancer, with consistent patterns observed across various tumor types, underscoring the need for cancer-specific monitoring strategies. This exhaustive analysis not only deepens our comprehension of the potential cardiac hazards associated with these therapeutic agents but also serves as a clarion call for clinicians to maintain heightened awareness and adopt proactive measures for early mitigation. Recognizing the exploratory essence of our work, we underscore the urgent need for a prospective validation study to fortify our discoveries. As we look ahead, we envision large-scale, population-centric prospective endeavors that will not only reveal the true magnitude of ADC-related AEs but also unravel the intricate biological pathways and predisposing factors, thereby paving the way for more effective risk assessment and management strategies.

## Supplementary Material

## Data Availability

ADC reports are available and can be retrieved form the FAERS Publish Dashboard (https://www.fda.gov/drugs/questions-and-answers-fdas-adverse-event-reporting-systemfaers/fdaadverse-event-reporting-system-faers-public-dashboard). The TCGA pan-cancer transcriptomic data are available and can be obtained from the UCSC Xena database (https://xenabrowser.net/datapages/).
