# Supplementary material for: Systematic analysis and mechanistic investigation of cardiac adverse events associated with antibody–drug conjugates using FAERS database
Source: Int J Surg. 2025 Sep 2;112(1):1436–47. doi: 10.1097/JS9.0000000000003314 (PMC12825936; doi:10.1097/JS9.0000000000003314)
Supplement: Supplementary file 2 [file js9-112-1436-002.docx]

Supplementary Table 3.

Clinical characteristics of reports with ADCs and ADCs & Dexamethasone from the FAERS database

| Characteristics | ADC-related cardiac AE reports (n = 2,361) | ADCs & Dexamethasone -related cardiac AE reports (n = 140) | *p* value |
| --- | --- | --- | --- |
| Gender, n (%) |  |  | 0.098 |
| Female | 1596(90.63%) | 96(89.72%) |  |
| Male | 165(9.37%) | 11(10.28%) |  |
| Age (years) |  |  | 0.7798 |
| n(Missing) | 1272(1089) | 97(43) |  |
| Mean±SD | 60.12±13.18 | 59.79±10.77 |  |
| Weight (kg), n (%) |  |  | 0.0253 |
| <80 | 477(78.84%) | 51(66.23%) |  |
| 80≤and≤100 | 98(16.20%) | 22(28.57%) |  |
| >100 | 30(4.96%) | 4(5.19%) |  |
