# Supplementary material for: Systematic analysis and mechanistic investigation of cardiac adverse events associated with antibody–drug conjugates using FAERS database
Source: Int J Surg. 2025 Sep 2;112(1):1436–47. doi: 10.1097/JS9.0000000000003314 (PMC12825936; doi:10.1097/JS9.0000000000003314)
Supplement: Supplementary file 3 [file js9-112-1436-003.docx]

**Cardiac Adverse Events Associated with ADCs in Breast Cancer Patients Reported in FAERS (2019–2023)**

1. **Scanning for ADC-related cardiac adverse events among ADC users in the FDA adverse events reporting system, 2019–2023**

To explore disease-specific patterns, we conducted a separate analysis of the breast cancer subgroup. In this population, the overall proportion of cardiac adverse events (cAEs) among ADC-related adverse events was 15.3%. A clear temporal decline was observed, with the proportion decreasing from 26.2% in 2019 to 11.1% in 2023 (Fig. 1A). This consistent downward trend within the breast cancer cohort suggests a stable temporal pattern of cAE reporting over time. In the breast cancer subgroup, the profile of cAEs at the preferred term (PT) level showed a consistent pattern with cardiotoxicity being the most frequently reported and strongest signal (ROR = 9.33) (Fig. 1B). Other notable events included cardiac dysfunction (ROR = 9.48), mitral valve incompetence (ROR = 6.90), and left ventricular dysfunction (ROR = 6.82), all of which exhibited strong disproportionality. Additional signals were observed for cardiac failure (ROR = 2.96), cardiomyopathy (ROR = 2.87), and pericardial effusion (ROR = 2.19).


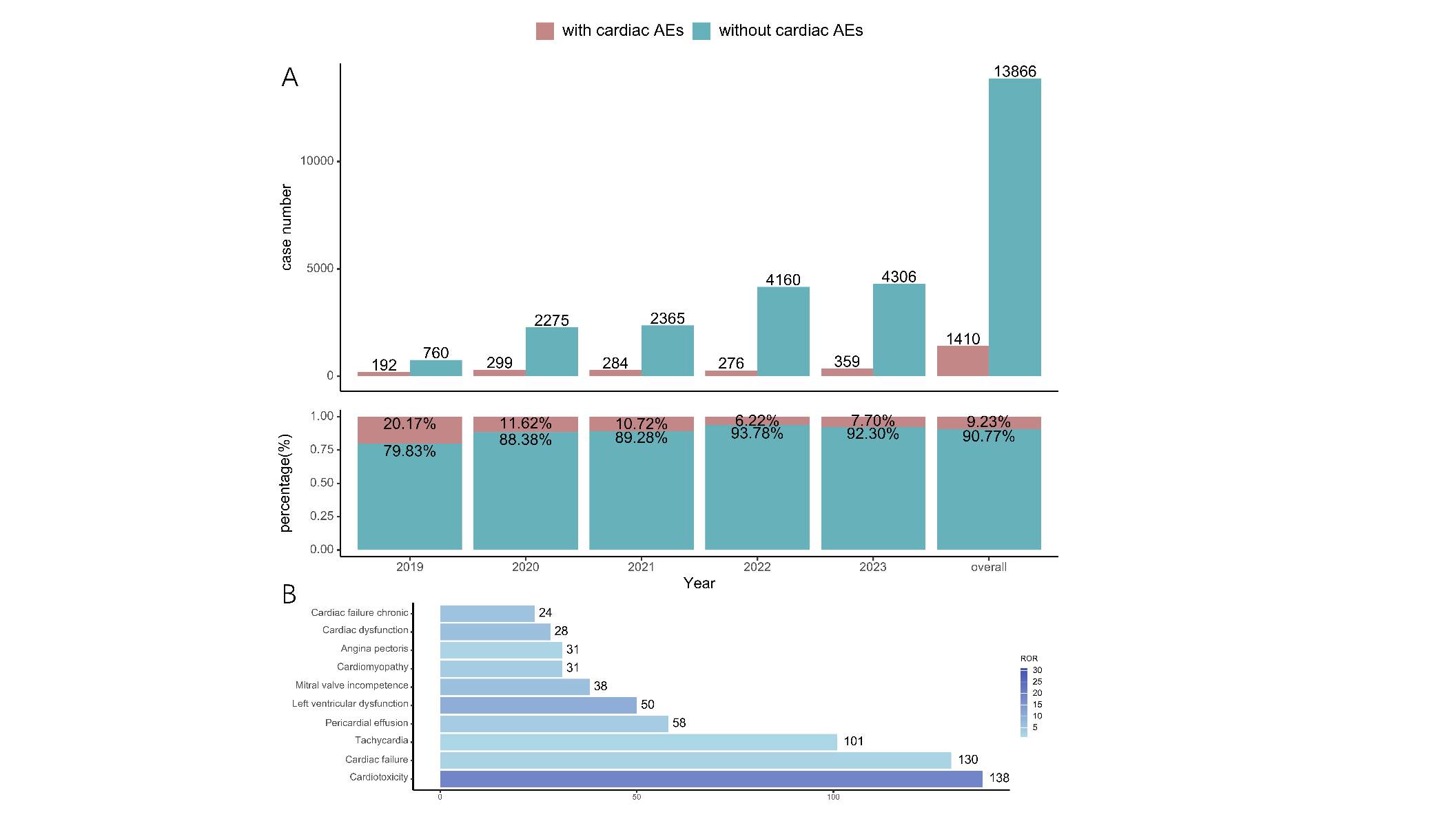


**Fig .1.** Overview of ADC-Associated Cardiac Adverse Events in FAERS Among Breast Cancer Patients (2019Q1–2023Q3). A) Bar plots showing the number and proportion of ADCs reports with vs. without cardiac adverse events. B) Top 10 most frequently reported cardiac adverse events across different ADC treatment strategies.

1. **Descriptive Analysis of ADC-Related Cardiac Adverse Events in Breast Cancer Patients**

The clinical characteristics of breast cancer patients experiencing ADC-related cAEs are summarized in Table 2. Compared to those with non-cardiac AEs, patients with cAEs were significantly older (mean age: 58.73 vs. 55.54 years, p < 0.0001) and showed distinct distributions in weight categories (p < 0.05). Due to the predominantly female composition of the breast cancer population, gender distribution did not differ between groups. However, significant differences were observed in clinical outcomes (p < 0.0001). Patients with cardiac AEs had a higher proportion of serious events, including a greater fatality rate (10.08% vs. 6.81%).

Table1.Clinical Characteristics of Breast Cancer Patients Treated with ADCs

| Characteristics | ADC-related cardiac AE reports (n = 1733) | ADC-related other AE reports (n = 13648) | *p* value |
| --- | --- | --- | --- |
| Gender, n (%) |  |  | 0.7525 |
| Female | 1307(98.72%) | 11013(98.82%) |  |
| Male | 17(1.28%) | 132(1.18%) |  |
| Age (years) |  |  | <0.0001 |
| n(Missing) | 939(794) | 7390(6258) |  |
| Mean±SD | 58.73±12.44 | 55.54±12.83 |  |
| Weight(kg), n (%) |  |  | <0.05 |
| <80 | 369(78.18%) | 3189(81.58%) |  |
| 80≤and≤100 | 83(17.58%) | 518(13.25%) |  |
| >100 | 20(4.24%) | 202(5.17%) |  |
| Outcomes, n (%) |  |  | <0.0001 |
| CA | 0(0.00%) | 4(0.03%) |  |
| DE | 118(6.81%) | 1376(10.08%) |  |
| DS | 32(1.85%) | 122(0.89%) |  |
| HO | 329(18.98%) | 2669(19.56%) |  |
| LT | 70(4.04%) | 200(1.47%) |  |
| OT | 1050(60.59%) | 6194(45.38%) |  |
| RI | 1(0.06%) | 10(0.07%) |  |
| Fatality, n (%) |  |  | <0.0001 |
| non-fatal | 1615(93.19%) | 12272(89.92%) |  |
| fatal | 118(6.81%) | 1376(10.08%) |  |

1. **Time-to-Onset of Cardiac Adverse Events Following ADC Use in Breast Cancer Patients**

In the breast cancer subgroup, over 50% of ADC-related cardiac adverse events (cAEs) occurred within 116 days of treatment initiation, and more than 80% occurred within 352 days (Fig. 2A). The median time to onset was 115.5 days (IQR: 91–138), indicating a generally delayed onset pattern compared to other populations (Fig. 2B).Stratification by sex showed a median onset of 116.5 days [92–138] in females and 169 days [64–NA] in males (Fig. 2C), although the difference was not statistically significant (p = 0.73), likely due to the limited number of male cases.Age-based analysis revealed no significant differences, but a trend toward longer onset in younger patients was observed. Patients under 65 years had a median onset of 118.5 days [102–149] versus 98 days [64–148] in those aged 65 or older (Fig. 2D, p = 0.523). When stratified by an alternative cutoff of 50 years, the median onset was 132 days [109–173] for patients aged ≤50 and 106 days [79–138] for those over 50 (p = 0.101) (Fig. 2E).

**
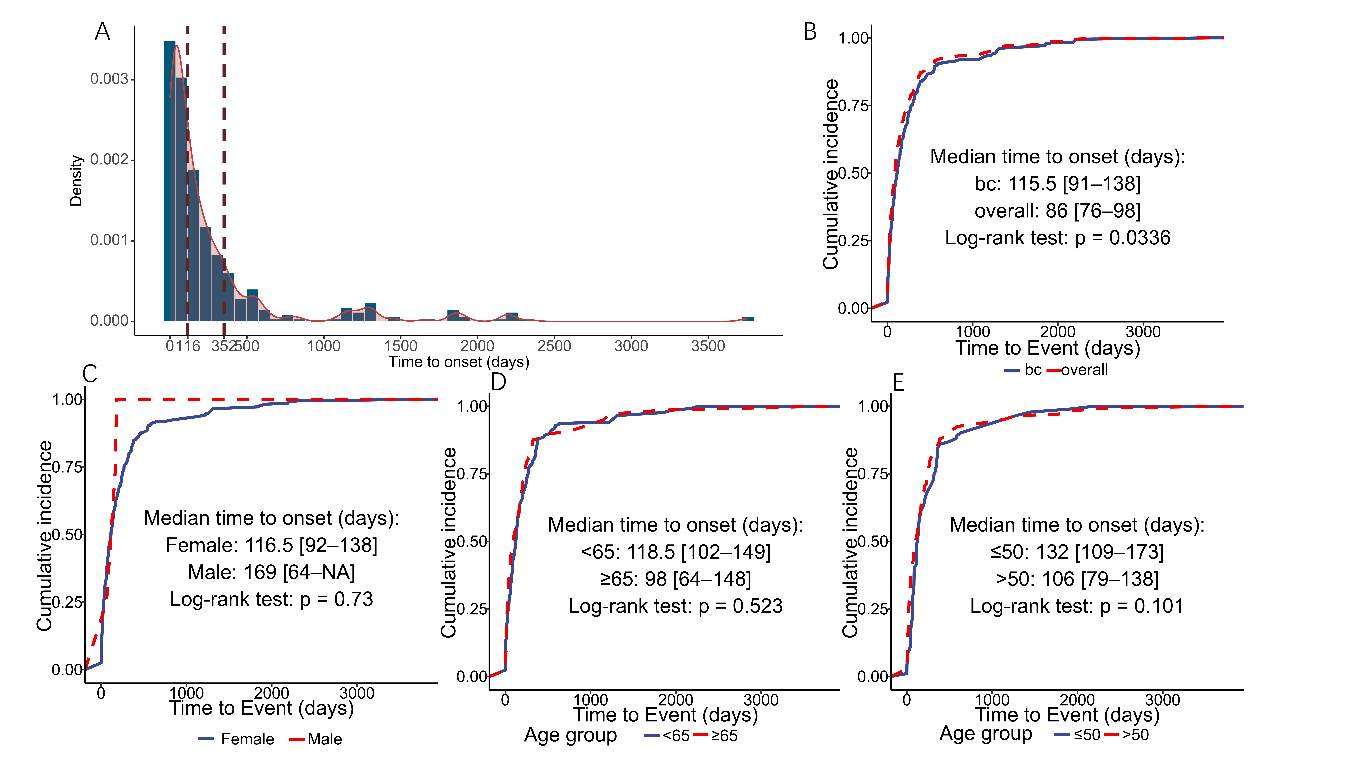
**

**Fig .2.** Time-to-onset analysis of cAEs in the breast cancer population. A) Cumulative distribution curve of overall onset time following ADC treatments. B) Cumulative distribution curve comparing overall time-to-onset between the breast cancer subgroup and the full cohort. C) Cumulative distribution curve of onset time stratified by gender. D, E) Cumulative distribution curves of onset time stratified by age using the 65-year cutoff (D) and the optimal 50-year cutoff (E).

1. **Risk Factors for ADC-Associated Cardiac Adverse Events in Breast Cancer Patients**

In the breast cancer subgroup, more than 50% of ADC-related cardiac adverse events (cAEs) occurred within 116 days of treatment initiation, and over 80% occurred within 352 days (Fig. 3A). The median time to onset was 115.5 days (IQR: 91–138), significantly longer than that observed in the overall cohort (86 days [76–98], p = 0.0336, log-rank test) (Fig. 3B).Sex-stratified analysis showed a median onset of 116.5 days [92–138] in female patients and 169 days [64–NA] in male patients (Fig. 7C), although this difference was not statistically significant (p = 0.73), likely due to the small number of male cases.Age-based comparisons showed no statistically significant differences. Patients under 65 years had a slightly longer median onset (118.5 days [102–149]) than those aged 65 and older (98 days [64–148], p = 0.523) (Fig. 3D). Similarly, using an alternative cutoff of 50 years, patients aged ≤50 years had a median onset of 132 days [109–173], compared to 106 days [79–138] in those >50 years (p = 0.101) (Fig. 3E).Collectively, these findings indicate a tendency toward delayed onset of cardiac AEs in breast cancer patients, underscoring the need for prolonged cardiac monitoring during ADC therapy in this population.

**
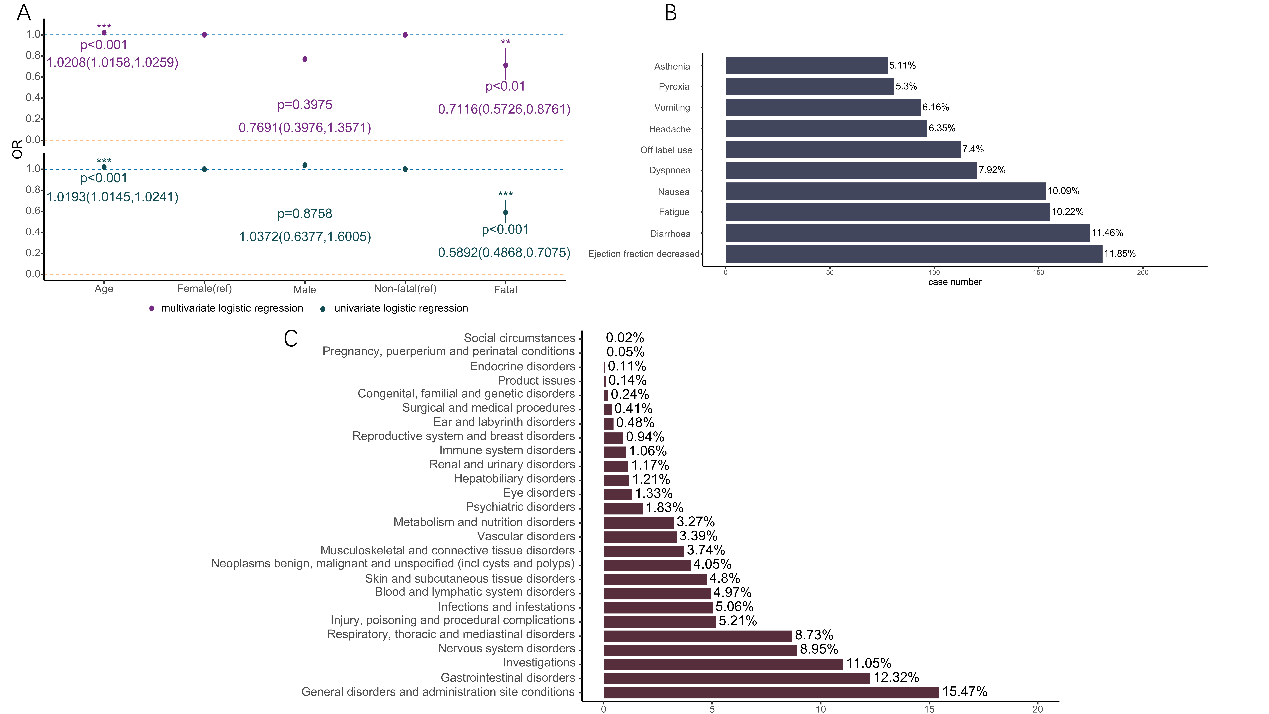
**

**Fig .3.** Time-to-onset analysis of cAEs in the breast cancer population. A) Cumulative distribution curve of overall onset time following ADC treatments. B) Cumulative distribution curve comparing overall time-to-onset between the breast cancer subgroup and the full cohort. C) Cumulative distribution curve of onset time stratified by gender. D, E) Cumulative distribution curves of onset time stratified by age using the 65-year cutoff (D) and the optimal 50-year cutoff (E).

1. **Cardiac Adverse Events in ADC Combination Therapy Among Breast Cancer Patients**

As shown in Fig. 4, co-administration of dexamethasone with ADCs in breast cancer patients was associated with a substantial reduction in the reporting odds ratios (RORs) for several cardiac adverse events. At the SOC level, the ROR for “cardiac disorders” decreased from 2.75 (95% CI: 2.62–2.88) with ADCs alone to 0.91 (95% CI: 0.70–1.07) when dexamethasone was co-administered.Similar reductions were observed at the PT level for key events, including cardiac failure, cardiomyopathy, pericardial effusion, and congestive cardiac failure. While some confidence intervals were relatively wide—likely due to limited sample sizes—the overall pattern aligns with the pan-cancer findings, suggesting that dexamethasone may exert a cardioprotective effect against ADC-related cAEs in breast cancer patients as well.

**
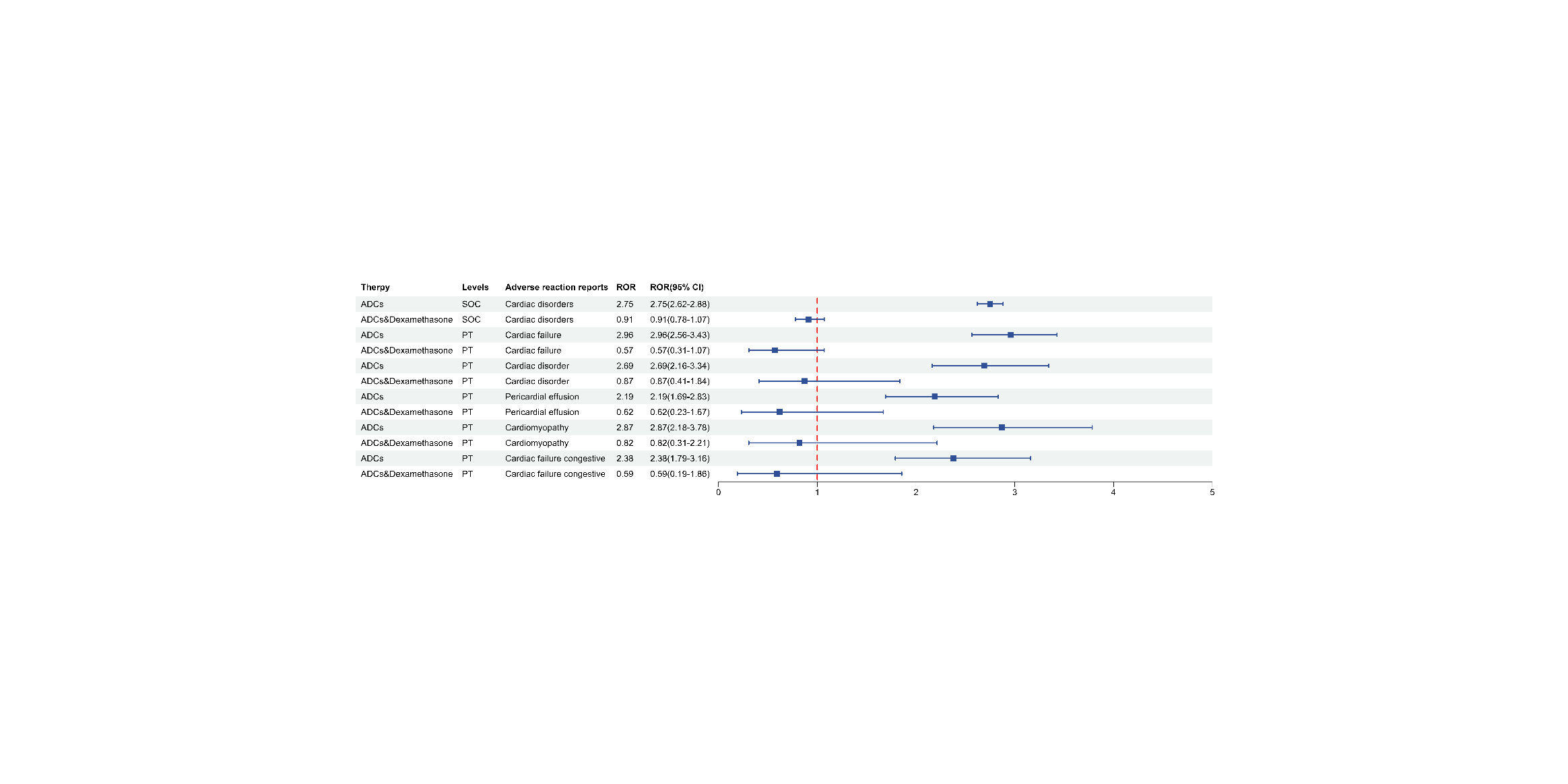
**

**Fig .4.** The signal distribution of ADCs alone and the combination of ADCs and dexamethasone in the breast cancer subgroup.

1. **Biological Mechanisms of ADC-Associated Cardiac Adverse Events in the breast cancer subgroup**

We did not perform pathway-level correlation analyses specifically for the breast cancer subgroup, as this cohort comprises a single cancer type with relatively limited transcriptomic heterogeneity. In contrast to the pan-cancer dataset, the absence of cross-cancer variation in gene expression among breast cancer cases constrains the interpretability of correlation-based pathway analyses. To avoid overinterpretation and maintain analytical rigor, we therefore elected not to repeat the transcriptomic correlation analysis within this subgroup.
