# Supplementary material for: Systematic analysis and mechanistic investigation of cardiac adverse events associated with antibody–drug conjugates using FAERS database
Source: Int J Surg. 2025 Sep 2;112(1):1436–47. doi: 10.1097/JS9.0000000000003314 (PMC12825936; doi:10.1097/JS9.0000000000003314)
Supplement: Supplementary file 4 [file js9-112-1436-004.docx]

**Cardiac Adverse Events Associated with ADCs in FAERS (2019–2023) Excluding Breast Cancer Cases**

1. **Scanning for ADC-related cardiac adverse events among ADC users in the FDA adverse events reporting system, 2019–2023**

After excluding breast cancer cases, the proportional burden of cardiac adverse events (cAEs) decreased but remained clinically relevant. The temporal pattern showed a steady decline over time, with cAEs accounting for 20.2% of ADC-related adverse events in 2019, decreasing to 11.6% in 2020, 10.7% in 2021, 6.2% in 2022, and 7.7% in 2023. Overall, the proportion of cAEs in the non–breast cancer population was 9.2% (Fig. 1A). The most frequently reported cAEs included cardiotoxicity (n = 138), which exhibited a high reporting odds ratio (ROR = 18.9). Other cardiac events with notable disproportionality included left ventricular dysfunction (ROR = 10.1), mitral valve incompetence (ROR = 6.08), cardiac dysfunction (ROR = 6.08), and chronic cardiac failure (ROR = 5.42). Interestingly, angina pectoris emerged as a new prominent cardiac adverse event (n = 31, ROR = 1.65) after exclusion of breast cancer cases. A detailed summary of patients experiencing angina pectoris following ADC treatment is provided in Supplementary Table 4. These results indicate that cardiac adverse events continue to represent a significant safety concern in the non–breast cancer population treated with ADCs.


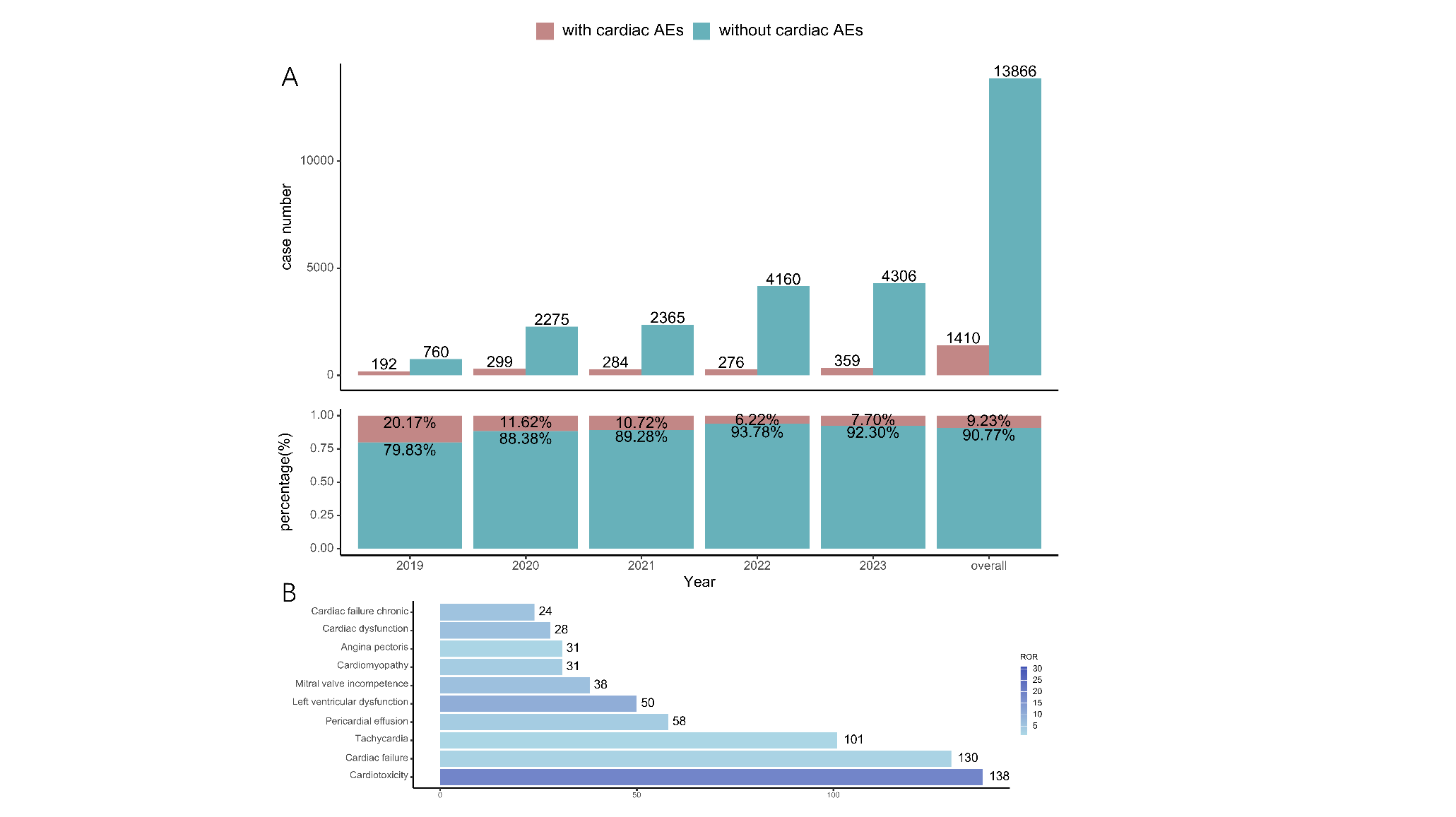


**Fig .1.** Overview of ADC-Associated Cardiac Adverse Events in FAERS After Excluding Breast Cancer (2019Q1–2023Q3). A) Bar plots showing the number and proportion of ADCs reports with vs. without cardiac adverse events. B) Top 10 most frequently reported cardiac adverse events across different ADC treatment strategies.

1. **Descriptive Analysis of ADC-Related Cardiac Adverse Events After Excluding Breast Cancer Cases**

We compared the demographic and clinical characteristics of ADC-related cardiac adverse event reports with other ADC-related adverse event reports in the non-breast cancer subgroup (Table 1). The data show that female predominance and age distribution were similar between groups. However, the fatality rate among cardiac AE reports was slightly higher in the non-breast cancer subgroup, though this difference did not reach statistical significance.

Table1. Clinical Characteristics of Non–Breast Cancer Patients Treated with ADCs in the FAERS Database

| Characteristics | ADC-related cardiac AE reports (n = 1045) | ADC-related other AE reports (n = 14321) | *p* value |
| --- | --- | --- | --- |
| Gender, n (%) |  |  | <0.0001 |
| Female | 590(78.88%) | 7029(65.93%) |  |
| Male | 158(21.12%) | 3625(34.00%) |  |
| UNK | 0(0.00%) | 8(0.08%) |  |
| Age (years) |  |  | 0.8061 |
| n(Missing) | 604(441) | 7125(7106) |  |
| Mean±SD | 61.77±13.53 | 61.91±13.86 |  |
| Weight(kg), n (%) |  |  | <0.05 |
| <80 | 223(74.83%) | 3030(82.00%) |  |
| 80≤and≤100 | 54(18.12%) | 430(11.64%) |  |
| >100 | 21(7.05%) | 235(6.36%) |  |
| Outcomes, n (%) |  |  | <0.0001 |
| CA | 0(0.00%) | 11(0.08%) |  |
| DE | 102(9.76%) | 1660(11.66%) |  |
| DS | 14(1.34%) | 103(0.72%) |  |
| HO | 207(19.81%) | 2972(20.88%) |  |
| LT | 32(3.06%) | 241(1.69%) |  |
| OT | 552(52.82%) | 4957(34.83%) |  |
| RI | 1(0.10%) | 15(0.11%) |  |
| Fatality, n (%) |  |  | 0.0629 |
| non-fatal | 943(90.24%) | 12571(88.34%) |  |
| fatal | 102(9.76%) | 1660(11.66%) |  |

1. **Time-to-Onset of Cardiac Adverse Events Following ADC Use, Excluding Breast Cancer Patients**

The cumulative probabilities of cAE onset reached 50% at 70 days and 80% at approximately 313 days (Fig. 2A). The median time to onset was 70 days [IQR: 54–91] (Fig. 2B). Sex subgroup analysis showed that males experienced significantly earlier onset than females (28 days [21–45] vs. 86 days [63–98], p < 0.0001) (Fig. 2C). Age stratification using a cutoff at 65 years revealed no significant difference (p = 0.0531) (Fig. 2D). However, using an optimal cutoff at 47 years demonstrated a significant difference in median time to onset: 77 days [45–124] for ≤47 years versus 62 days [42–87] for >47 years (p = 0.0434) (Fig. 2E).

**
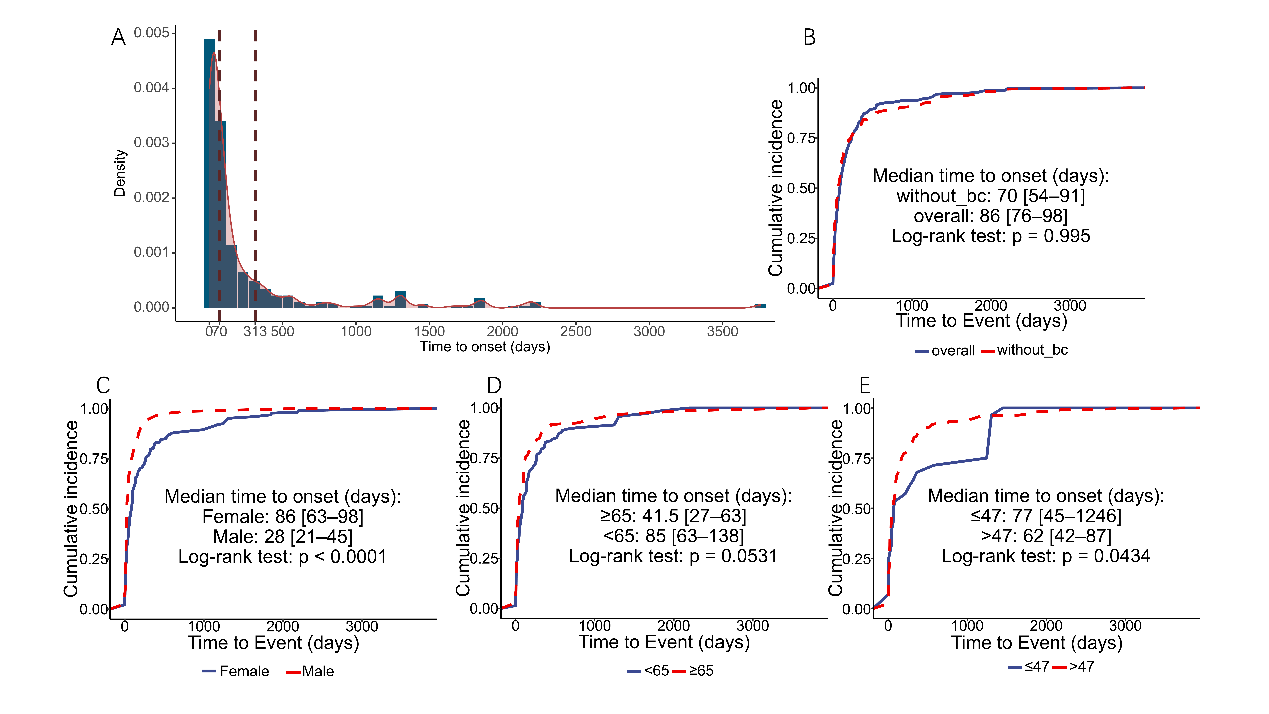
**

**Fig .2.** Time-to-onset analysis of cAEs in the non-breast cancer population. A) Cumulative distribution curve of overall onset time following ADC treatments. B) Cumulative distribution curve comparing overall time-to-onset between the non-breast cancer subgroup and the full cohort. C) Cumulative distribution curve of onset time stratified by gender. D, E) Cumulative distribution curves of onset time stratified by age using the 65-year cutoff (D) and the optimal 47-year cutoff (E).

1. **Risk Factors for ADC-Associated Cardiac Adverse Events After Excluding Breast Cancer Cases**

Univariate logistic regression showed that age was not a significant factor for cAE risk (OR = 0.9997, 95% CI: 0.9947–1.0049, p = 0.9235). Male gender was significantly associated with a reduced risk (OR = 0.5413, 95% CI: 0.4659–0.6267, p < 0.001), while fatal events were less likely to be reported as cAEs compared to non-fatal events (OR = 0.7314, 95% CI: 0.5972–0.8880, p < 0.05). In multivariate analysis, male gender retained its protective effect (OR = 0.5189, 95% CI: 0.4320–0.6207, p < 0.001). Age became statistically significant (OR = 1.0058, 95% CI: 1.0002–1.0115, p < 0.05), suggesting a modest independent influence on cAE risk. Fatality was no longer significant after adjustment (OR = 0.9209, 95% CI: 0.7358–1.1415, p = 0.4612) (Fig. 3A).

At the system organ class (SOC) level, the most frequently reported concomitant AEs were general disorders and administration site conditions (14.58%), gastrointestinal disorders (12.03%), and investigations (10.42%) (Fig. 3B). At the preferred term (PT) level, diarrhea (7.47%), nausea (7.07%), off-label use (7.07%), fatigue (6.88%), and dyspnea (6.09%) were among the top reported events (Fig. 3C).

**
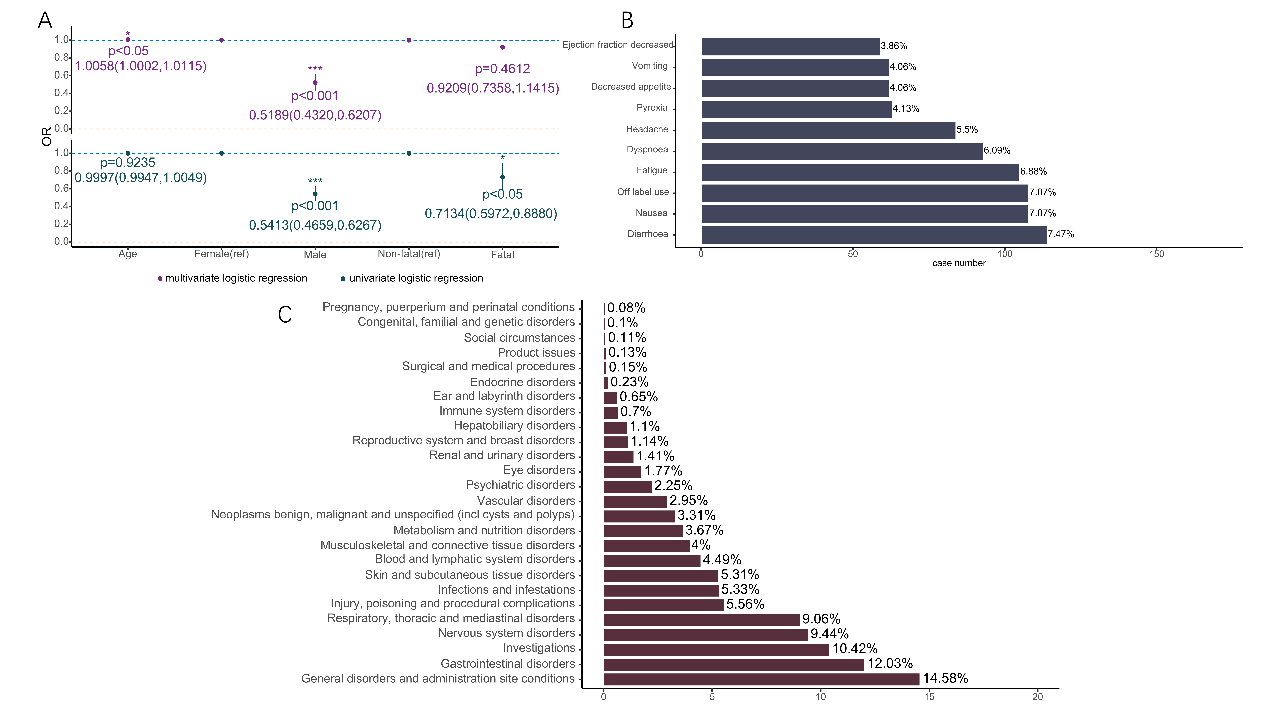
**

**Fig .3.** Analysis of factors and co-reported adverse events associated with ADC-related cAEs in the non-breast cancer subgroup. A) Forest plot of univariate (green) and multivariate (purple) logistic regression results for factors associated with ADC-related cAEs. OR indicates odds ratio. *P < 0.05; **P < 0.01; ***P < 0.001. B) Distribution of co-reported adverse events by SOC. C) Top 10 PTs of co-reported adverse events.

1. **Cardiac Adverse Events in ADC Combination Therapy Excluding Breast Cancer Patients**

The updated disproportionality analysis showed that co-administration of dexamethasone was associated with reduced reporting odds ratios (RORs) for cardiac adverse events related to ADCs (Fig. 4). At the system organ class (SOC) level, the ROR for cardiac disorders decreased from 1.38 with ADCs alone to 0.80 when combined with dexamethasone. At the preferred term (PT) level, reductions were observed for cardiac failure (ROR 2.11 to 0.79), cardiac disorder (1.16 to 0.60), and congestive cardiac failure (1.14 to 0.76). Although RORs for pericardial effusion and cardiomyopathy also decreased (from 3.44 to 1.81 and from 3.45 to 2.72, respectively), the confidence intervals were wide, reflecting variability due to limited sample sizes. Overall, these findings support a protective effect of dexamethasone on ADC-related cardiac adverse events in the non-breast cancer population, suggesting the potential generalizability of this effect across a broader oncologic cohort.

**
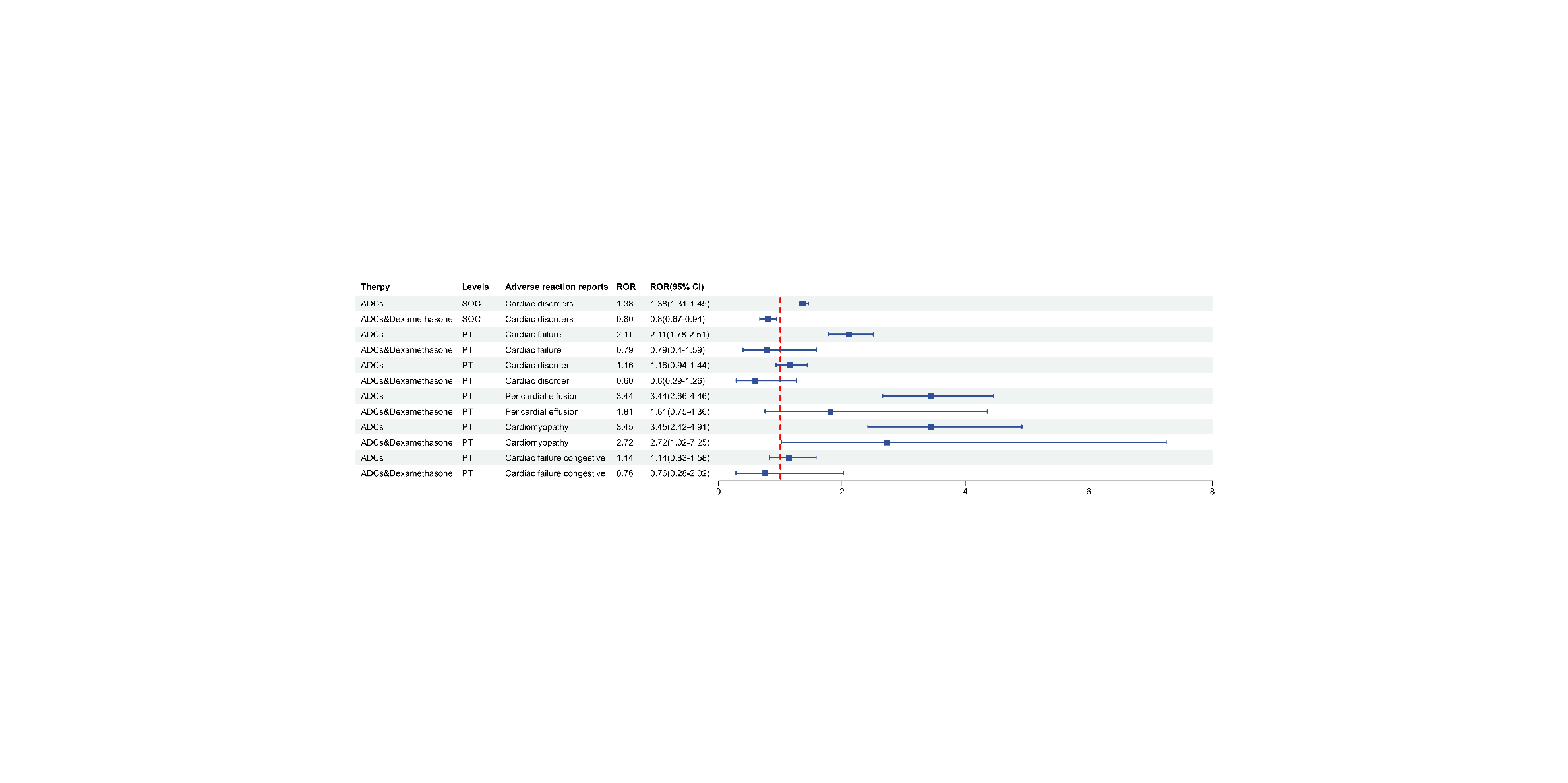
**

**Fig .4.** The signal distribution of ADCs alone and the combination of ADCs and dexamethasone after excluding breast cancer cases.

1. **Biological Mechanisms of ADC-Associated Cardiac Adverse Events Excluding Breast Cancer Cases**

After excluding breast cancer cases, the correlation patterns between ADC-related cardiac adverse event (cAE) signals and transcriptome features across TCGA pan-cancers remained largely consistent. A significant negative correlation persisted with the hepatocyte growth factor receptor signaling pathway (R = -0.72, p = 5.93e-03) and calcium-dependent cysteine-type endopeptidase activity (R = -0.67, p = 1.27e-02). Positive correlations with heat shock protein binding (R = 0.66, p = 1.42e-02) and HSP70 protein binding (R = 0.61, p = 2.72e-02) were also maintained, albeit with slight attenuation. These results indicate that the molecular pathways associated with ADC-related cAEs remain relevant across multiple tumor types beyond breast cancer.

**
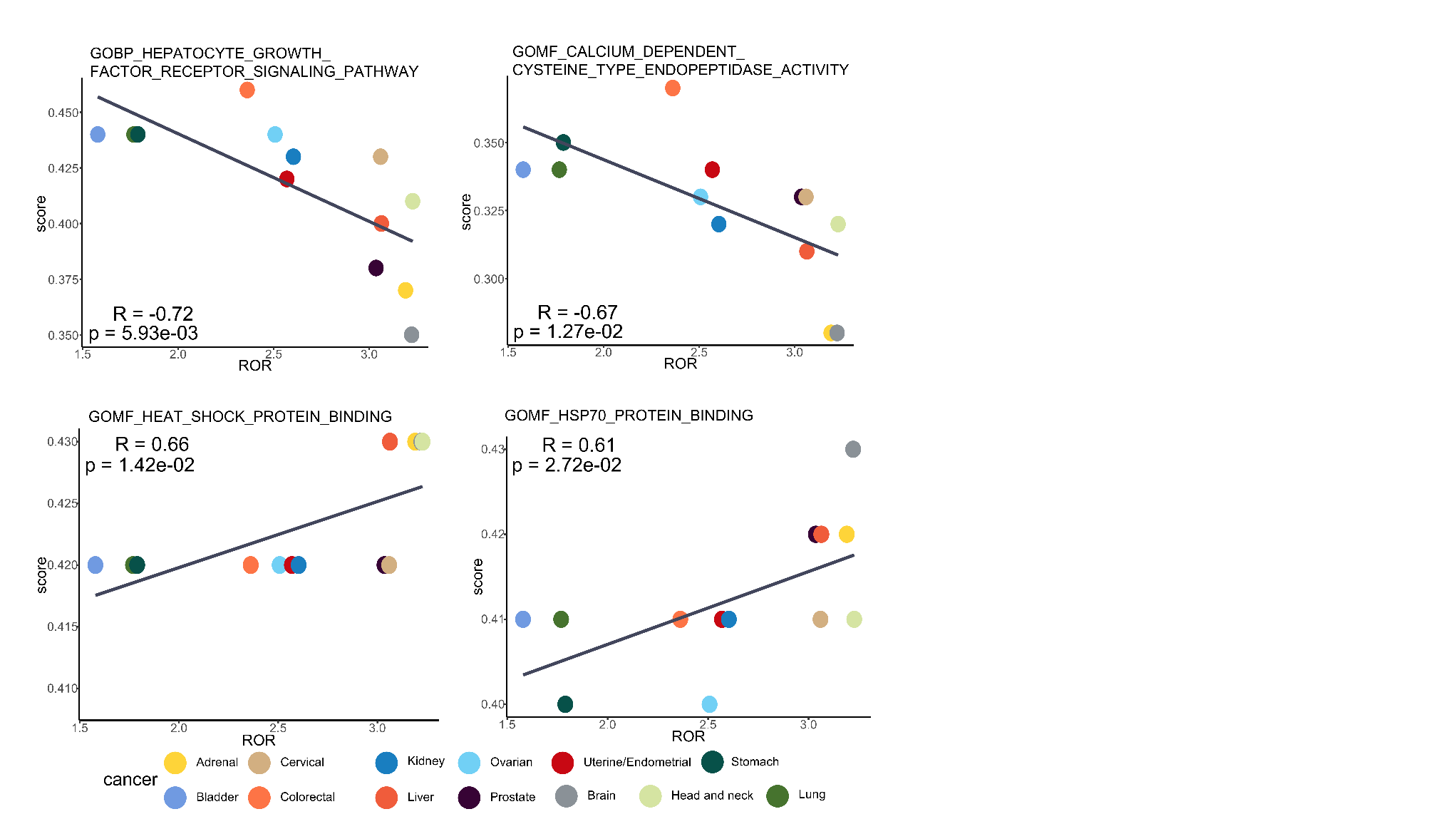
**

**Fig .5.** Correlation between RORs of ADC-related cAEs and ssGSEA pathway enrichment scores after excluding breast cancer cases, analyzed using Spearman’s rank correlation.
