# Supplementary material for: Systematic analysis and mechanistic investigation of cardiac adverse events associated with antibody–drug conjugates using FAERS database
Source: Int J Surg. 2025 Sep 2;112(1):1436–47. doi: 10.1097/JS9.0000000000003314 (PMC12825936; doi:10.1097/JS9.0000000000003314)
Supplement: Supplementary file 6 [file js9-112-1436-006.docx]

Detailed Information of Angina Pectoris Patients after Excluding Breast Cancer Cohort

| Case id | Age | Gender | Weight (Kg) | Drug name | outcome | GetDataYear |
| --- | --- | --- | --- | --- | --- | --- |
| 15595785 | 61 | F | 80 | TRASTUZUMAB | HO | 19Q3 |
| 16055569 | 64 | F | NA | HERCEPTIN | OT | 19Q1 |
| 16526248 | NA | NA | NA | TRASTUZUMAB | OT | 20Q2 |
| 16721853 | 62 | F | 80 | TRASTUZUMAB | NA | 23Q3 |
| 16828701 | 61 | F | 80 | TRASTUZUMAB | OT | 20Q1 |
| 16989632 | 61 | F | 80 | TRASTUZUMAB | HO | 20Q1 |
| 17029260 | NA | F | NA | HERCEPTIN | OT | 19Q4 |
| 17203389 | 52 | F | 70 | TRASTUZUMAB | HO | 19Q4 |
| 17597236 | NA | F | NA | TRASTUZUMAB | OT | 20Q1 |
| 18313516 | NA | F | 106 | HERCEPTIN | OT | 22Q2 |
| 18527051 | 60 | F | 80 | ADO TRASTUZUMAB EMTANSINE | HO | 21Q4 |
| 18569061 | 61 | F | 80 | TRASTUZUMAB | HO | 22Q1 |
| 19106963 | NA | NA | NA | HERZUMA | HO | 21Q4 |
| 19167227 | NA | NA | NA | TRASTUZUMAB | NA | 23Q3 |
| 19493122 | NA | NA | NA | TRASTUZUMAB | HO | 21Q4 |
| 19986892 | NA | F | NA | TRASTUZUMAB | OT | 21Q4 |
| 20012986 | NA | F | NA | TRASTUZUMAB | OT | 21Q4 |
| 20055483 | NA | NA | NA | TRASTUZUMAB | OT | 21Q4 |
| 20141061 | NA | F | NA | TRASTUZUMAB | OT | 21Q4 |
| 20637246 | 76 | F | 52.3 | TRASTUZUMAB | HO | 22Q1 |
| 20638348 | 76 | F | 52.3 | TRASTUZUMAB | HO | 22Q1 |
| 20714484 | 76 | F | 52.3 | TRASTUZUMAB | HO | 22Q2 |
| 20876360 | 81 | M | NA | PADCEV | NA | 23Q3 |
| 21280371 | NA | F | 78 | HERCEPTIN | OT | 22Q3 |
| 21362787 | 50 | F | NA | TRASTUZUMAB | OT | 22Q3 |
| 21365895 | 50 | F | NA | TRASTUZUMAB | HO | 22Q3 |
| 21419859 | NA | NA | NA | TRASTUZUMAB | HO | 22Q4 |
| 21471485 | 77 | F | NA | TRASTUZUMAB | HO | 22Q4 |
| 22726659 | NA | NA | NA | TRASTUZUMAB | NA | 23Q3 |
| 22747858 | NA | F | 80 | TRASTUZUMAB | NA | 23Q3 |
| 22755631 | 53 | F | 68.04 | OGIVRI | NA | 23Q3 |
